# Repertory to the Symptoms of Intermittent Fever

**Published:** 1883-05

**Authors:** 


REPERTORY TO THE SYMPTOMS OF INTERMITTENT FEVER. By William A. Allen, M. D. Pp. 107. Philadelphia: Hahnemann Publishing House, F. E. Bœricke. 1883.

Intermittent fever is to most homœopathists a very difficult subject for treatment. We are convinced that this trouble lays more with the physician than with any inherent obstinacy in the disease. We are all too prone to prescribe for "intermittent fever" and not for the peculiar symptoms presented by the patient. That is, we prescribe for those symptoms which are common to all cases of the disease, thus unwisely ignoring those characteristic of the case before us. Those physicians who prescribe homœopathically declare they find no difficulty in curing intermittent fever with the dynamized drugs. Dr. Allen adds his testimony to this effect. Dr. H. C. Allen some time ago gave us an excellent monograph on the therapeutics of this disease; Dr. William Allen now supplements that work with a repertory. Those who have much to do with this disease owe these gentlemen an eternal debt of gratitude for their excellent work.

We feel that we can safely recommend Dr. Allen's Repertory to our friends. It is newer than Bœnninghausen's though not so full and complete.

